# Seroreactivity of the Severe Acute Respiratory Syndrome Coronavirus 2 Recombinant S Protein, Receptor-Binding Domain, and Its Receptor-Binding Motif in COVID-19 Patients and Their Cross-Reactivity With Pre-COVID-19 Samples From Malaria-Endemic Areas

**DOI:** 10.3389/fimmu.2022.856033

**Published:** 2022-04-27

**Authors:** Abdouramane Traoré, Merepen A. Guindo, Drissa Konaté, Bourama Traoré, Seidina A. Diakité, Salimata Kanté, Assitan Dembélé, Abdourhamane Cissé, Nathan C. Incandela, Mamoudou Kodio, Yaya I. Coulibaly, Ousmane Faye, Andrey V. Kajava, Federico Pratesi, Paola Migliorini, Anna Maria Papini, Lorenzo Pacini, Paolo Rovero, Fosca Errante, Mahamadou Diakité, Myriam Arevalo-Herrera, Socrates Herrera, Giampietro Corradin, Saidou Balam

**Affiliations:** ^1^ Immunogenetic Laboratory and Parasitology, University of Sciences, Techniques and Technologies of Bamako (USTTB), Bamako, Mali; ^2^ Department of Ministry of Health and Social Development, Hopital de Dermatologie de Bamako (HDB), Bamako, Mali; ^3^ Center for Polymers and Organic Solids, Department of Chemistry and Biochemistry, University of California Santa Barbara, Santa Barbara, CA, United States; ^4^ Montpellier Cell Biology Research Center (CRBM), University of Montpellier, CNRS, Montpellier, France; ^5^ Immuno-Allergology Unit, Department of Clinical and Experimental Medicine, University of Pisa, Pisa, Italy; ^6^ Interdepartmental Research Unit of Peptide and Protein Chemistry and Biology, Department of Chemistry “Ugo Schiff”, University of Florence, Florence, Italy; ^7^ Interdepartmental Research Unit of Peptide and Protein Chemistry and Biology, Department of Neurosciences, Psychology, Drug Research and Child Health, Section of Pharmaceutical Sciences and Nutraceutics, University of Florence, Florence, Italy; ^8^ Department of Immunology, Malaria Vaccine and Drug Development Center, Cali, Colombia; ^9^ Department of Immunology, Caucaseco Scientific Research Center, Cali, Colombia; ^10^ Biochemistry Department , University of Lausanne, Lausanne, Switzerland; ^11^ Department of Nephrology, University Hospital Regensburg, Regensburg, Germany

**Keywords:** SARS-CoV-2 S protein, seroreactivity, COVID-19 samples, cross-reactivity, Pre-COVID-19 samples, malaria endemic-area

## Abstract

Despite the global interest and the unprecedented number of scientific studies triggered by the COVID-19 pandemic, few data are available from developing and low-income countries. In these regions, communities live under the threat of various transmissible diseases aside from COVID-19, including malaria. This study aims to determine the severe acute respiratory syndrome coronavirus 2 (SARS-CoV-2) seroreactivity of antibodies from COVID-19 and pre-COVID-19 samples of individuals in Mali (West Africa). Blood samples from COVID-19 patients (n = 266) at Bamako Dermatology Hospital (HDB) and pre-COVID-19 donors (n = 283) from a previous malaria survey conducted in Dangassa village were tested by ELISA to assess IgG antibodies specific to the full-length spike (S) protein, the receptor-binding domain (RBD), and the receptor-binding motif (RBM_436–507_). Study participants were categorized by age, gender, treatment duration for COVID-19, and comorbidities. In addition, the cross-seroreactivity of samples from pre-COVID-19, malaria-positive patients against the three antigens was assessed. Recognition of the SARS-CoV-2 proteins by sera from COVID-19 patients was 80.5% for S, 71.1% for RBD, and 31.9% for RBM (*p* < 0.001). While antibody responses to S and RBD tended to be age-dependent, responses to RBM were not. Responses were not gender-dependent for any of the antigens. Higher antibody levels to S, RBD, and RBM at hospital entry were associated with shorter treatment durations, particularly for RBD (*p* < 0.01). In contrast, higher body weights negatively influenced the anti-S antibody response, and asthma and diabetes weakened the anti-RBM antibody responses. Although lower, a significant cross-reactive antibody response to S (21.9%), RBD (6.7%), and RBM (8.8%) was detected in the pre-COVID-19 and malaria samples. Cross-reactive antibody responses to RBM were mostly associated (*p* < 0.01) with the absence of current *Plasmodium falciparum* infection, warranting further study.

## Introduction

Coronaviruses are a group of enveloped viruses containing a single-stranded RNA genome with positive polarity (1). They include severe acute respiratory syndrome coronavirus (SARS-CoV), Middle East respiratory syndrome (MERS-CoV) (1–3), and severe acute respiratory syndrome coronavirus 2 (SARS-CoV-2) or COVID-19 (4, 5). COVID-19 affects people of all ages, but morbidity and mortality are more significant in the elderly and those with chronic diseases (6–9). Emerging in China in 2019 (10), COVID-19 rapidly spread worldwide and was declared a pandemic by the WHO in March 2020^1^ ^2^. More than 200 million cases and over 4 million deaths have been reported worldwide, affecting 220 countries and territories^3^, generating massive economic and social consequences. The first COVID-19 case diagnosed in Mali was reported on March 25, 2020, and Malian health authorities quickly established a strategy to control the disease^4^. In addition, the authorities have promoted the harmonization of research activities by leveraging research laboratory capacities and strengthening relationships among local and international stakeholders (11–13).

Despite considerable global efforts to study the immune responses elicited by SARS-CoV-2 and their role in clinical protection and pathogenesis (14–17), the host factors leading to low or moderate clinical manifestations, as well as completely asymptomatic infections, are not well understood. Initial analysis indicates that certain populations have been exposed to other microorganisms, either pathogenic or non-pathogenic, which appear to induce immune responses against COVID-19 (i.e., antibodies or potentially other immune effectors that contribute to reducing or preventing COVID-19 clinical manifestations (18–25)).

Specific antibody responses to COVID-19 have been reported in moderately and severely symptomatic SARS-CoV-2-positive individuals (26–32). However, there are few data available linking symptomatic disease and duration of hospitalization or treatment with specific antibodies to SARS-CoV-2 antigens. Such antibodies may be detected as early as the end of the first week of illness; however, they may also take weeks to appear, giving rise to different clinical outcomes (29, 33). In addition, the presence or absence of protective immunity due to infection or vaccination may affect future transmission and disease severity (29).

Of notable importance, it has been observed that there are significantly lower COVID-19 clinical cases and fatalities in malaria-endemic regions than in non-endemic areas (19, 22, 34). Several host factors, including sociodemographic conditions, genetic background, and immune status, could be influencing the COVID-19 clinical evolution. Moreover, other SARS cases, induced by viruses potentially sharing common immunodominant antigens, might affect the outcome of the disease (18, 20, 35, 36).

Considering the burden of malaria in Mali (37) and the potential for clinical overlap with COVID-19, efforts to both study diseases and understand the potential immunological interplay are ongoing (19, 22). This potential relationship has tremendous epidemiological relevance not only for understanding clinical outcomes in malaria-endemic and non-endemic regions but also for COVID-19 vaccination efforts. In the absence of a specific anti-SARS-CoV-2 treatment, research into this area is of considerable importance.

The spike (S) protein is encoded by a systematic interplay between the SARS-CoV-2 genome, the nucleocapsid (N), the membrane (M), the envelope (E), and various additional structural proteins. It plays a crucial role in viral infection and pathogenesis of COVID-19 (38, 39), as it is essential for the viral invasion of the host cell, mainly through its RBD domain (5, 9, 37). Both RBD and its ligand, the human angiotensin-converting enzyme-2 (ACE2), are crucial research targets for developing COVID-19 therapeutic antibodies, vaccines, and serological tests (2, 40–45). Currently, most COVID-19 vaccines in use or development are based on the S protein; however, the different vaccine platforms have demonstrated a variety of strengths and weaknesses^5^. In addition to the commonly used S protein and its RBD, we designed (manuscript submitted) and studied the S protein’s receptor binding motif (RBM_436–507_) that interacts with ACE2.

Vaccine success is likely associated with the specificity and strength of the immune response it triggers against the S protein, specifically against its RBD. However, this immune response may also correlate with factors like age, gender, ethnicity, disease experience (i.e., disease evolution), treatment duration, and comorbidities, among others (6–8).

In light of all these issues, this study aimed to assess the natural antibody response specific to the full-length S protein, its functional domains RBD (protein), and RBM (peptide) using plasma collected from COVID-19-positive patients and pre-COVID-19 participants from a malaria-endemic region. The epidemiological paradox observed in COVID-19 and malaria patients in the initial phase, and in the dynamics of infection in malaria-endemic countries (19, 22), promotes the need for further studies in this area to produce a better understanding of the genetic and immunological factors involved.

## Methods

### Study Type, Periods, and Sites

A cross-sectional study was conducted to assess the seroreactivity of COVID-19 patients and pre-COVID-19 donors against the SARS-CoV-2 full-length recombinant S protein and its binding domains RBD and RBM. Samples were collected from the Dermatology Hospital of Bamako (HDB) in Mali (West Africa); sociodemographic and epidemiological surveys were also carried out. While all COVID-19 blood samples were collected from patients confirmed to harbor SARS-CoV-2 by RT-PCR test, pre-COVID-19 plasma samples were gathered in 2019—before the onset of the COVID-19 pandemic—and therefore were not tested by COVID-19 RT-PCR. The latter were collected from donors living in the Village of Dangassa in Mali, a malaria-endemic zone, and were stored frozen at −20°C. All laboratory tests were performed at the Laboratory of Immunogenetic and Parasitology, at the International Centre of Excellence in Research (ICER-Mali) of the University of Sciences, Techniques and Technologies of Bamako (Mali). The data management and sample processing were carried out from May 2021 to September 2021.

### Study Population

The study population included COVID-19-infected patients (n = 266; sex *ratio = 1.2 in favor* of men) with SARS-CoV-2 confirmed by RT-PCR and admitted to the HDB for inpatient care. The pre-COVID-19 population consisted of volunteers (n = 283; sex *ratio = 1.1 in favor* of women) who had participated in a previous malaria survey study in 2019, before the onset of COVID-19 in Mali. The study population (COVID-19 and pre-COVID-19 participants) were stratified by age groups 1–4, 5–9, 10–14, 15–19, 20–29, 30–39, 40–49, 50–59, 60–69, and 70+ years. This adjusted for the age structure of the population as recommended by the WHO guidelines on population-based sero-surveys of SARS-CoV-2 infection^6^. COVID-19 participants provided sociodemographic and epidemiological data, including comorbidities and length of treatment duration. Pre-COVID-19 participants had records of sociodemographic and epidemiological data, and current *Plasmodium falciparum* infection (parasitemia) was confirmed by microscopic examination after Giemsa staining of blood smear (BS) slides. None of the participants had a history of COVID-19 vaccination.

### Ethical Considerations

This study was approved by the Institutional Review Board (Ethics Committee, EC) of the Faculties of Medicine and Odontostomatology and of the Pharmacy of Bamako (with reference N°2021/25/CE/USTTB). Written informed consent (IC) was obtained from each COVID-19 patient for the collection of blood samples, sociodemographic information, and clinical data for future investigative purposes. The authorization of the use of pre-COVID-19 samples and data was also obtained from the same EC and under the reference cited above. The current study was based on available data from participants whose plasma samples and related data were available and accessible. The confidentiality of the participants’ data was preserved throughout this study.

### Variables, Data, and Sample Collections

Data analysis was carried out using medical records from the HDB data register. Data were collected at the time of hospital admission (on week 1) and during hospitalization at HDB in 2020. Data were collected using a paper questionnaire developed for this purpose, including 1) sociodemographic information; 2) symptoms and severity of disease; 3) comorbidities or factors such as diabetes, hypertension, asthma, and body weight; 4) clinical evolution of the disease’s form; and 5) duration of hospital stay or treatment. The pre-COVID-19 participant samples were collected from the village of Dangassa in 2019 before the onset of COVID-19 in Mali. The variables in the pre-COVID-19 group included sociodemographic (age and gender) and epidemiological data such as the presence and density of current *P. falciparum* infection. A BS slide was performed and examined by microscopy for the presence and density of *P. falciparum* [positive (BS+) or negative (BS−) for each pre-COVID-19 sample].

Whole blood (5–10 ml) was collected from each COVID-19 patient by venipuncture upon admission to HDB, and the sample transportation to the laboratory was carried out following the WHO guidelines for Infectious Substances 2019–2020 (46). Trained biologists were responsible for ensuring compliance with these guidelines.

### Protein Sequence Analysis, Design, and Antigen Production

Sequences of the S protein were downloaded from the National Center for Biotechnology Information (NCBI) SARS-CoV-2 Resources^7^. Recombinant proteins from the full-length S and RBD were provided by ExcellGene SA (Monthey, Switzerland) and Protein Production and Structure Core Facility, EPFL (Lausanne, Switzerland)^8^. Proteins were produced according to the manufacturer’s recommendations^9^. A peptide covering the receptor-binding interface (receptor binding motif, RBM_436–507_) of the S protein was synthesized at the Chemistry Department, Florence University, Florence, Italy. RBM is known to undergo some post-translational modifications (PTMs) such as glycosylation, but this does not directly contribute to the binding affinity between SARS-CoV-2 S and ACE-2 (47). In addition, as it is a synthetic product used in ELISA, RBM is not expected to undergo any further modification. The 3D images were generated using PyMol software, an open-source molecular graphics tool (48) using the atomic coordinates from PDB entry 6ZOY (49). The illustrative diagram of domains, amino acid sequences, and the 3D structure of the S protein displaying both the RBD and RBM sequences are all shown in **Supplementary Figure 1**.

### Enzyme-Linked Immunosorbent Assay

Sample seroreactivity was studied using an ELISA with 96-well plates (type of plate, Ref 442404). Plates were coated with 1 μg/ml of S, RBD, or RBM (antigen coating) or not coated with an antigen (non-antigen coating) and then incubated overnight (O/N) at 4°C. The plates were then blocked for 1 h at room temperature (RT) with phosphate-buffered saline (PBS) 1× (3% milk) before being incubated for 2 h at RT with COVID-19 and pre-COVID-19 plasma samples at a dilution of 1:100. Goat anti-human IgGs, conjugated to horseradish peroxidase (HRP), were used as secondary antibodies, diluted to 1:5,000 (Life Technologies, Carlsbad, CA, USA; Ref H10307), and incubated for 1 h at RT. Signals were revealed using TMB substrate reagent (BD OptEIA, cat 555214; BD Biosciences, San Jose, CA, USA) for 20 min in the dark at RT, and the reaction was stopped using 1 M of sulfuric acid (Merck, Darmstadt, Germany; 1.00731.1000). Optical density (OD) was measured at 450/630 nm in a microplate ELISA-Reader (SoftMax^®^Pro Software). Samples were considered positive when their mean OD was ≥mean OD + 3SD of the negative control samples (indicated as the cutoff). The cross-reactivity of pre-COVID-19 samples was considered significant for the samples with a mean OD ≥ mean OD + 3SD of the negative controls with a dilution of 1:100 (indicated as the cutoff). Non-specific binding samples (i.e., samples with antibody responses in non-antigen-coated plates), were determined to be samples with an OD against non-coated plates greater or equal to the same sample’s response against antigen-coated plates (i.e., responder sample).

### Data Management and Statistical Analysis

Data from the coded questionnaires were directly entered into the electronic data entry system during data and sample collection. Each participant was assigned a number that was known only to the investigators. The information was entered in Excel 2013, and ELISA data were imported directly into Excel and associated with the participants’ sociodemographic and epidemiological data. The analysis and generation of figures were done with Stata and Prism 5 software. The unpaired t-test, chi-squared test, and Fisher’s exact test were used to compare groups with a significance threshold of 5%.

## Results

### Sequences and 3D Structures of S Protein, and the Receptor-Binding Domain and Receptor-Binding Motif Domains

Three antigens, namely, the full-length S protein (1250 aa), its RBD (211 aa), and a synthetic peptide covering the binding interface (RBM; 72 aa) of RBD, were used in this study (**Supplementary Figure 1**). The S protein plays a crucial role in viral infection and pathogenesis, as it mediates the SARS-CoV-2 binding to human ACE2. It comprises two functional subunits: S1, which harbors the N-terminal domain (NTD) and the receptor-binding domain (RBD), responsible for binding to the host cell receptor; and the S2, which harbors the heptad repeat 1 (HR1) and 2 (HR2), responsible for the fusion of viral and cell membranes (39) (**Supplementary Figure 1A**). The full-length sequence of the S protein of SARS-CoV-2 was obtained using the BLASTP search program (50, 51). The SARS‐CoV‐2 RBD shows significant sequence homology (~73%) with seasonal phylogenetically related coronaviruses (25, 52–54) (**Supplementary Figure 1B**). The RBM is a segment representing approximately 6% of the S protein’s length, located within the RBD domain. It is recognized by the ACE2 protein and not only represents the most variable region of the protein but is also highly specific to SARS-CoV-2 (**Supplementary Figure 1C**). The 3D image of the SARS-CoV-2 S protein structure was made while displaying the RBD and RBM locations (48, 49) (**Supplementary Figure 1D**).

### Seroprevalence of Antibodies Against S, Receptor-Binding Domain, and Receptor-Binding Motif in COVID-19 Patients

**Figure 1:**
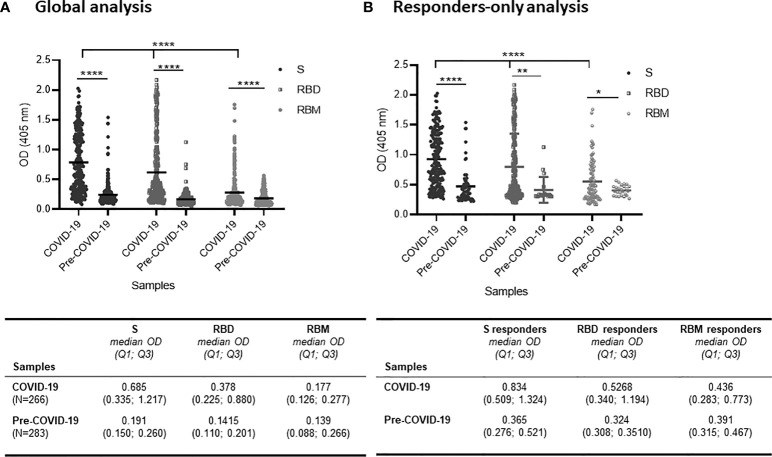
Distribution of antibody responses against S, receptor-binding domain (RBD), and receptor-binding motif (RBM) antigens in COVID-19 and pre-COVID-19 samples. **(A)** Global analysis of samples (positive and negative in ELISA) shows that antibody (Ab) levels (mean OD shown as a horizontal black line in the dot plots) for S, RBD, and RBM were significantly higher in COVID-19 patient samples as compared to pre-COVID-19 donor samples (*p* < 0.0001). Also, the Ab levels varied significantly (*p* < 0.0001) among S, RBD, and RBM in COVID-19 samples. The table shows the median OD, Q1, and Q3 values of antibodies for S, RBD, and RBM in COVID-19 and pre-COVID-19 samples. **(B)** Levels of Ab responses in responder-only COVID-19 samples were significantly higher than in responder-only pre-COVID-19 samples (cross-reactive responders) for S (*p* < 0.0001), RBD (*p* < 0.01), and RBM (*p* < 0.05). The table shows the median OD, Q1, and Q3 of antibodies for S, RBD, and RBM of responder-only samples in COVID-19 and pre-COVID-19 participants. The unpaired t-test and ANOVA were performed to compare the mean ODs of antibodies between the two groups and within the groups themselves, respectively. *p < 0.05; ** *p* < 0.01; ****p < 0.0001; OD, optical density; Q1, quartile 1; Q3, quartile 3.

Overall, all three antigens were well recognized by the COVID-19 samples but with significant variation among the S, RBD, and RBM antigens (*p* < 0.0001; **Figure 1A**). In terms of antibody prevalence, of the 266 samples studied, 214 samples (80.5%) recognized S, 189 (71.1%) recognized RBD, and 85 (31.9%) recognized RBM (**Table 1**). In terms of antibody level, the S protein showed a two-fold higher antibody OD than RBD, which in turn showed a two-fold higher antibody OD than RBM; the median OD and interquartile 1 and 3 (Q1; Q3) were 0.685 (0.335; 1,217), 0.378 (0.225; 0.880), and 0.177 (0.126; 0.277), respectively (**Figure 1A**).

**Table 1:** Frequency of responders against S, RBD, and RBM in COVID-19 and pre-COVID-19 donors.

Samples	S responder *n (%)*	RBD responder *n (%)*	RBM responder *n (%)*
***COVID-19 (N = 266)***	214 (80.5)	189 (71.1)	85 (31.9)
***Pre-COVID-19 (N = 283)***	62 (21.9)	19 (6.7)	25 (8.8)
*p*	**	**	**

The proportion of responder samples against S, RBD, and RBM was calculated using the samples showing an antibody mean OD ≥ mean OD + 3SD of the negative controls at the dilution 1:100 (indicated as the ELISA cutoff). Fisher’s exact test was used to compare the proportion of responders between the COVID-19 and pre-COVID-19 groups. N, total number of samples; n, number of responder samples; %, percent of responder samples; RBD, receptor-binding domain; RBM, receptor-binding motif; OD, optical density.

**p ≤ 0.01.

**Figure 2:**
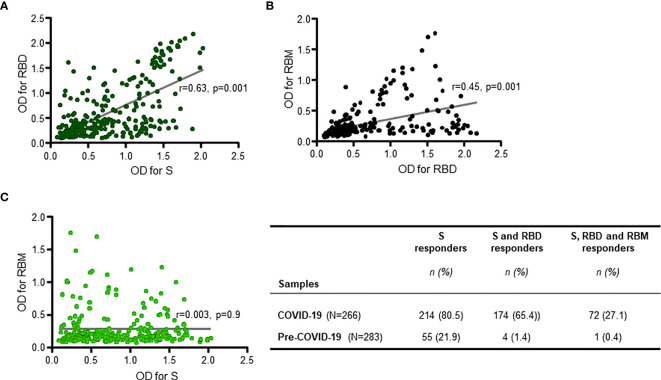
Positive responder samples from COVID-19 patients simultaneously recognizing two or all three antigens. There was a significant positive correlation between antibody responses (antibody optical density (OD)) against S and receptor-binding domain (RBD) (R = 0.63, *p* = 0.001 **(A)**), and between antibody responses against RBD and receptor-binding motif (RBM) ((R = 0.45, *p* = 0.001 **(B)**), but not for antibody responses against S and RBM (R = 0.003, *p* = 0.9 **(C)**). The two-sided Spearman’s rank correlation test was used to determine *p*- and R-values. The gray lines are the lines of best fit for each scatter diagram. The table shows the number (n) and prevalence (%) of responder samples recognizing only S, or only S and RBD, or recognizing all three antigens simultaneously.

**Figure 3:**
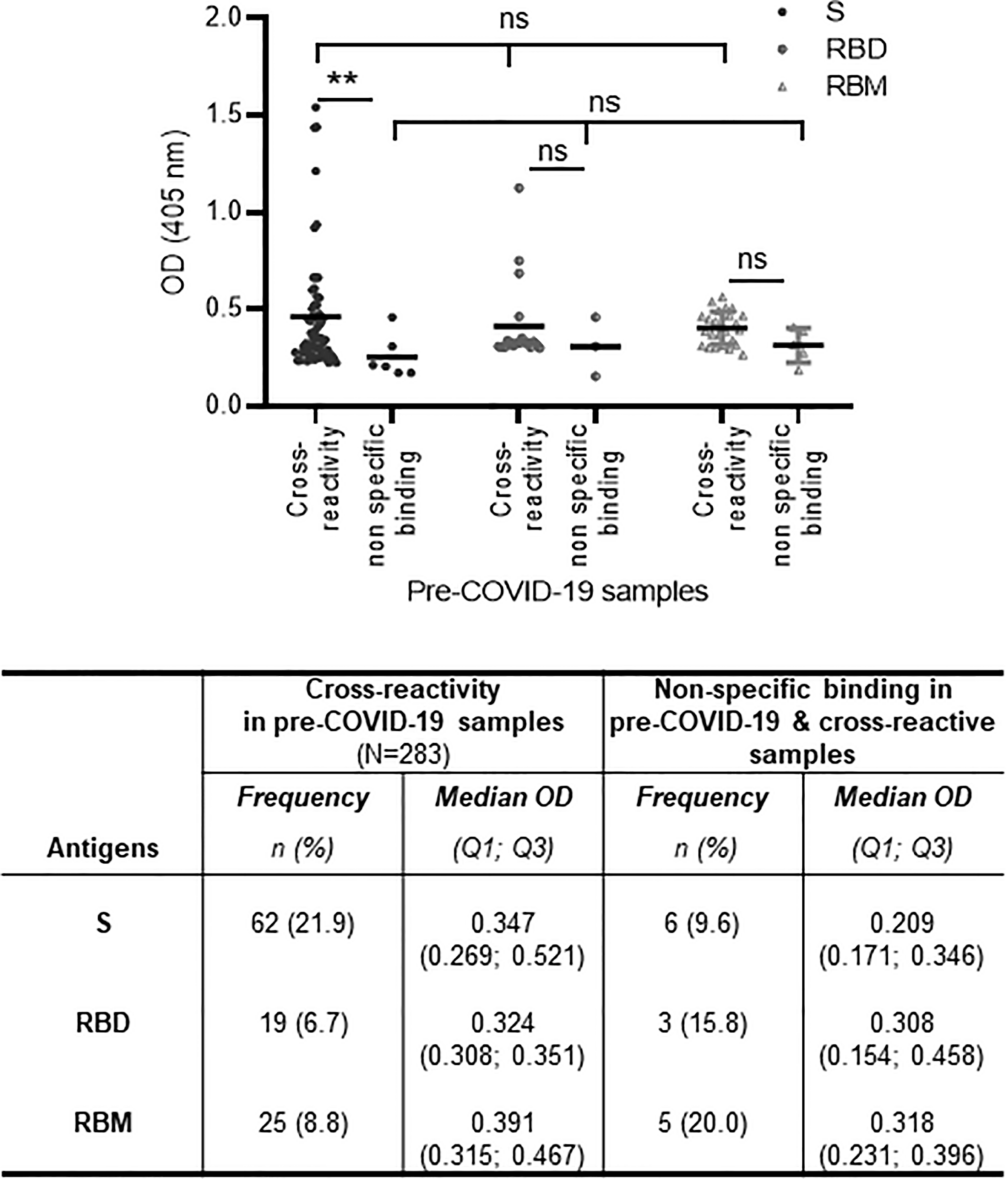
Cross-reactivity and non-specific binding against S, receptor-binding domain (RBD), and receptor-binding motif (RBM) in pre-COVID-19 and endemic malaria samples. The cross-reactive antibody levels (mean optical density (OD) shown as a horizontal black line in the dot plots) for S, RBD, and RBM were demonstrably higher than in non-specific binding antibody levels; this was significant for S (*p* < 0.01). The table shows the number and proportion (frequency) of samples showing cross-reactions or non-specific binding for S, RBD, and RBM. N, total number of pre-COVID-19 samples; n, number of cross-reactive or non-specific binding samples; %, percent of cross-reactive or non-specific binding samples; Q1, quartile 1; Q3, quartile 3. The unpaired t-test and ANOVA were used to compare mean antibody ODs between different groups and within the groups themselves, respectively. **p ≤ 0.01; ns, not significant.

When only the reactive samples (responders) were assayed, the S protein showed a higher median OD for Q1 and Q3 [0.834 (0.509; 1.324)] than did RBD [0.5268 (0.340; 1.194)] or RBM [0.436 (0.283; 0.773)] (**Figure 1B**). While reactivity with S and RBD was observed in 65.5% (174/266), only 27.1% (72/266) of COVID-19 donors recognized all three antigens (**Figures 2A**–**C**). This reactivity would be relevant in selecting antibody donors and antigens for further analysis. The recognition of S correlated with recognition of RBD (r = 0.63, *p* = 0.001; **Figure 2A**), and recognition of RBD correlated with recognition of RBM (r = 0.45, *p* = 0.001; **Figure 2B**). In contrast, there was little correlation between the recognition of S and the recognition of RBM (r = 0.003, *p* = 0.9; **Figure 2C**). Although samples from pre-COVID-19 volunteers (n = 283) presented lower reactivity frequencies and ODs than the COVID-19 samples (*p* < 0.05; **Figures 1**, **2**; **Table 1**), they still displayed a significant level of cross-reactivity against the three antigens (see **Figure 3** and **Supplementary Figure 4**).

The analysis of IgG antibody levels by gender (male (M) and female (F)) in the COVID-19 patient group indicated comparable results between the two genders for each antigen (**Supplementary Figures 2A–C**). In the COVID-19 patient group, the median OD (Q1; Q3) for M vs. F was 0.609 (0.333; 1.260) vs. 0.712 (0.339; 1.193), 0.371 (0.229; 0.873) vs. 0.390 (0.213; 0.887), and 0.174 (0.130; 0.251) vs. 0.186 (0.123; 0.300) for S, RBD, and RBM, respectively (**Supplementary Figures 2A–C**). The frequency of responders and antibody OD were both similar between M and F (*p* > 0.05) in both COVID-19 and pre-COVID-19 groups, except for the cross-reactive response to RBM (**Supplementary Figure 2C**) in the pre-COVID-19 group (**Table 2**). Furthermore, the non-specific binding of antibody samples in COVID-19 patients accounted for 8.9% (17 out of 189), and 14.1% (12 out of 85) of the seroreactive samples for S, RBD, and RBM, respectively (**Table 3**).

**Table 2:** Prevalence of antibody responders against S, RBD, and RBM according to gender in COVID-19 and pre-COVID-19 sample groups.

	S responders			RBD responders			RBM responders		
Samples	*Male n (%)*	*Female n (%)*	*p*	*Male n (%)*	*Female n (%)*	*p*	*Male n (%)*	*Female n (%)*	*p*
***COVID-19 (N = 266)***	116 (79.5)	98 (81.7)	*ns*	105 (71.9)	84 (70.0)	ns	44 (30.1)	41 (34.2)	ns
***Pre-COVID-19 (N = 283)***	32 (23.7)	30 (20.3)	ns	7 (5.2)	12 (8.1)	ns	7 (5.2)	18 (12.1)	*

The proportions of S, RBD, and RBM responders in COVID-19 samples as compared to pre-COVID-19 samples were determined. Fisher’s exact test was used to compare the proportion of responders between COVID-19 and pre-COVID-19 samples.

N, total number of samples; n, number of responders; %, percentage of responders; RBD, receptor-binding domain; RBM, receptor-binding motif; ns, not significant.

^*^p ≤ 0.05.

**Table 3:** Proportion of non-specific binding antibodies against S, RBD, and RBM responders in COVID-19 patients.

COVID-19 samples (N = 266)				
Antigens	*Responders*		*Non-specific Ab binding from responders*	
	*n (%)*	*Median OD (Q1; Q3)*	*n (%)*	*Median OD (Q1; Q3)*
***S***	214 (80.5)	0.834 (0.509; 1.324)	19 (8.9)	0.664 (0.504; 0.781)
***RBD***	189 (71.1)	0.527 (0.340; 1.194)	17 (8.9)	0.728 (0.626; 0.884)
***RBM***	85 (31.9)	0.436 (0.283;0.773)	12 (14.1)	0.737 (0.642; 0.866)

The proportion of samples showing non-specific binding antibodies for S, RBD, and RBM was determined in COVID-19 patient samples. The non-specific binding antibody samples are those showing in no antigen-coating, i.e., in plates coated with no antigen, a mean OD of antibody ≥ mean OD in antigen coating. The median OD and interquartile (Q1 and Q3) are illustrated.

N, number of COVID-19 samples; n, number of responders or non-specific binding samples, %, the proportion of responders or non-specific binding samples; RBD, receptor-binding domain; RBM, receptor-binding motif.

**Figure 4:**
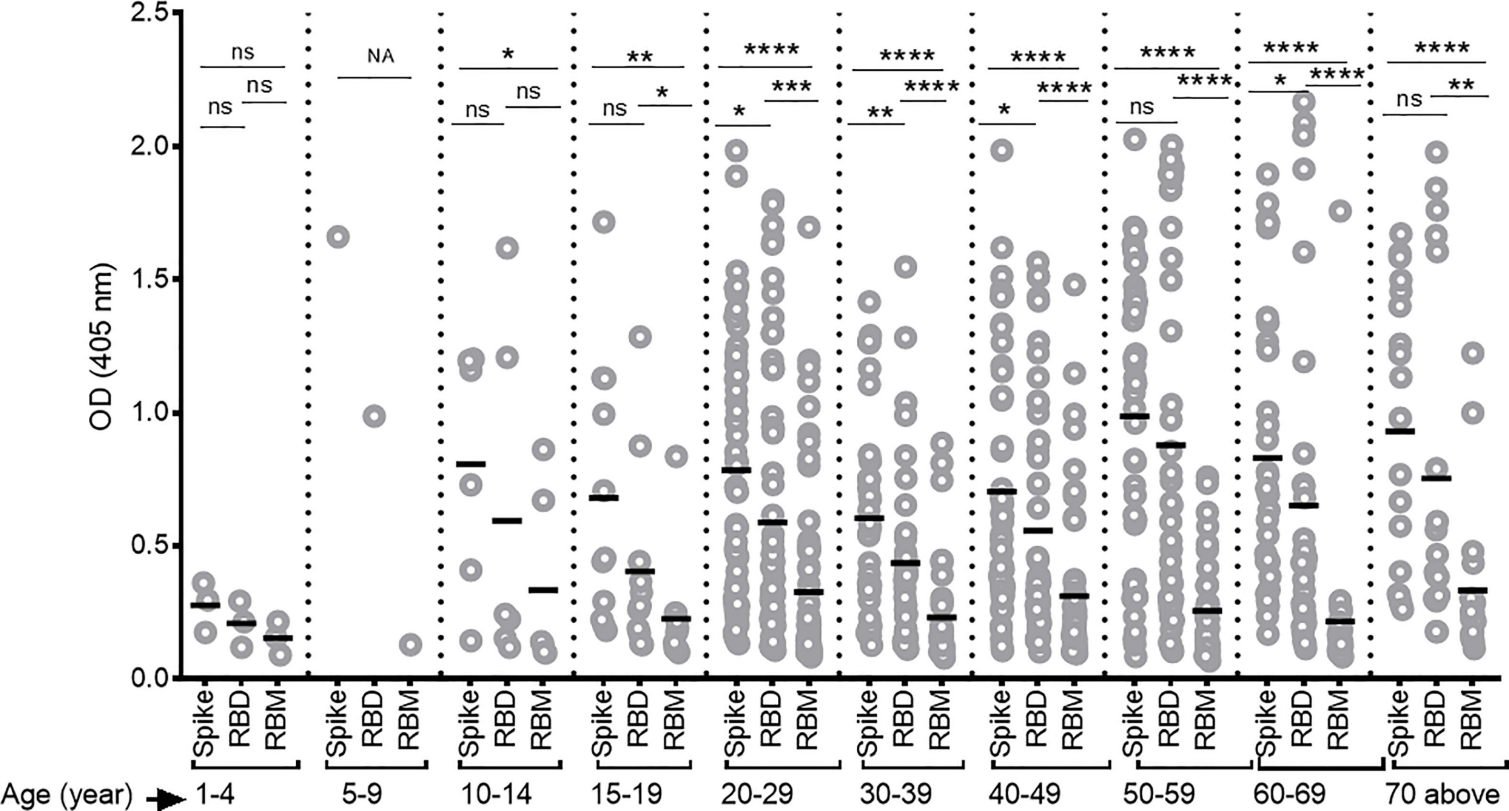
Differing antibody responses against S, receptor-binding domain (RBD), and receptor-binding motif (RBM) according to different age groups of COVID-19 patients. Antibody responses against S, RBD, and RBM were studied for each age group of COVID-19 patients. A correlation was observed between increasing antibody levels and increasing age. The average Ab response (mean optical density (OD)) against each antigen was calculated for each age group. Comparisons were made using an unpaired t-test to study the difference in responses against each antigen within each age group. NA, not applicable; *p < 0.05; **p < 0. 01; ***p < 0.001; ****p < 0.0001. ns, not significant; Age (year), age ranges in years.

Overall, antibody levels increased as a function of age—particularly for S and RBD—but not for the RBM fragment (**Figure 4**). Furthermore, antibody levels to S and RBD were comparable at the earlier ages under 19 and above 59 years and were significantly greater than those against RBM.

### Levels of Anti-S, Receptor-Binding Domain, and Receptor-Binding Motif Antibodies at Hospital Admission and Duration of Remission From the Symptomatic COVID-19

**Figure 5:**
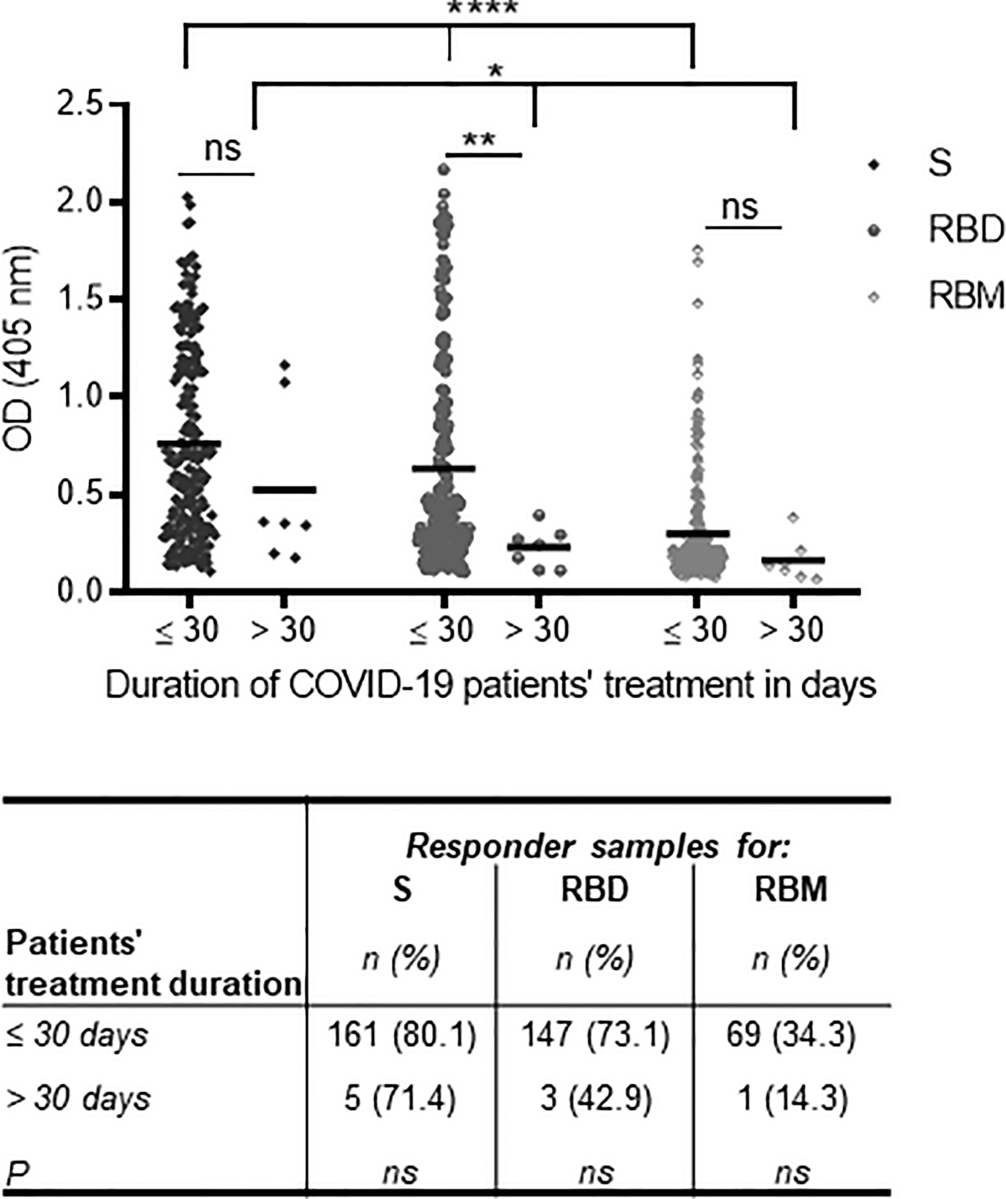
Association of anti-S, receptor-binding domain (RBD), and receptor-binding motif (RBM) antibodies at the time of hospital admission with the duration of treatment for symptomatic COVID-19. The lowest antibody levels for S, RBD, and RBM at the time of hospital admission were associated with increased patient treatment time for symptomatic forms of COVID-19 (i.e., >30 days) as shown in the graph. The correlation was strongest with RBD recognition. The table shows the proportions of S, RBD, and RBM responders as a function of their treatment duration, but no significant difference was observed between the three antigens and the treatment duration time. The unpaired t-test and ANOVA were used to compare the mean Ab optical density (OD) between the two treatment duration groups and between antigens, respectively, and Fisher’s exact test was used to determine the proportion of responders with a treatment duration of ≤30 or >30 days. *p < 0.05; **p < 0.01; ****p < 0.0001. ns, not significant.

Here, we analyze the association between antibody levels toward S, RBD, and RBM at the time of hospital admission and duration of treatment (i.e., the remission of symptomatic forms). Duration of remission was thus defined as the estimated time in days (≤30 or >30 days) from hospital admission to recovery from symptomatic SARS-CoV-2 infections, as confirmed by at least two negative RT-PCRs. Overall, the duration of treatment was shorter for participants who had higher antibody levels at admission for all three antigens, especially for RBD (*p* < 0.01) (**Figure 5**). In addition, for the patient group with treatment periods ≤30 days, Ab levels for S, RBD, and RBM varied more significantly from each other (*p* < 0.0001) than among those hospitalized for longer periods (*p* = 0.037) (**Figure 5**). However, the proportion of responder samples for S, RBD, or RBM was comparable between the ≤30- and >30-day treatment groups (**Figure 5**).

### Preexisting Comorbid Conditions and Elicitation of Anti-S, Receptor-Binding Domain, and Receptor-Binding Motif Antibodies Among COVID-19 Patients

**Figure 6:**
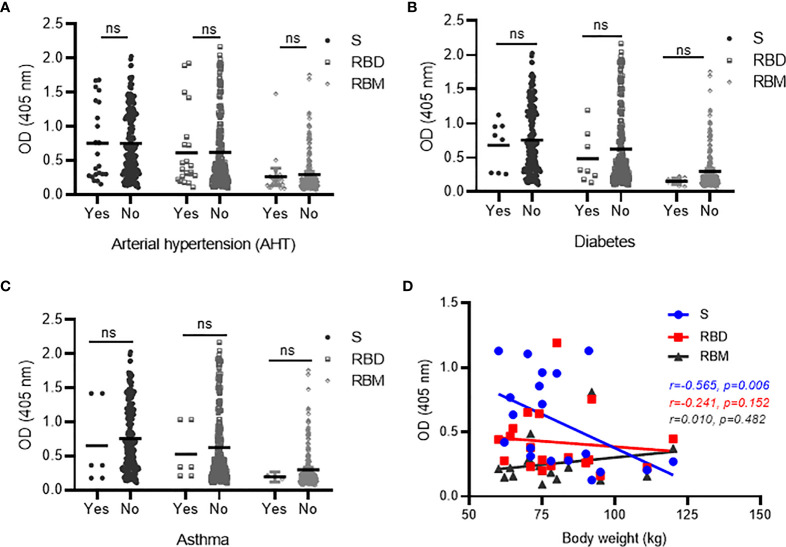
Correlation between antibodies against S, receptor-binding domain (RBD), and receptor-binding motif (RBM) and comorbid conditions in COVID-19 patients. No significant variation was observed in antibody responses against S, RBD, or RBM between COVID-19 patients with the presence (Yes) vs. absence (No) of comorbid conditions, such as AHT (hypertension) **(A)**, diabetes **(B)**, and asthma **(C)**. However, a trend toward increased antibody levels for all three antigens was observed in the COVID-19 patient groups with no diabetes **(B)** or asthma **(C)**. **(D)** Spearman’s rank analysis shows a significant negative correlation between antibody levels for S and body weight but showed no significant impact on antibodies against RBD and RBM in COVID-19 patients. ns, not significant.

Comorbidities such as diabetes, hypertension and asthma, and high body weight were evaluated as factors that may impact the effective development of antibodies against S, RBD, and RBM in COVID-19 patients. The antibody levels (mean OD) for S, RBD, and RBM were similar between the patient groups with and without arterial hypertension (AHT) and were slightly higher in the patient groups not suffering from diabetes or asthma (**Figures 6A**–**C**). Similarly, the prevalence of antibody responders for S and RBD remained similar between patient groups with or without comorbidity (*p* > 0.05; **Table 4**), whereas COVID-19 patient groups suffering from asthma and diabetes showed no positive antibody responses against RBM (**Table 4**). In addition, increasing body weight was associated with a significant decrease in antibody responses to S and a slight decline in antibody response to RBD (**Figure 6D**). The occurrence of two or more simultaneous comorbidities in a COVID-19 patient did not significantly impact the level of anti-S- and RBD-specific antibodies; however, there was no correlation between two comorbidities in COVID-19 patients and the response against RBM (**Supplementary Figures 3A–C**).

**Table 4:** Proportion of antibody responders for S, RBD, and RBM in conjunction with the presence or absence of comorbid conditions among COVID-19 patients.

	S responders		RBD responders		RBM responders	
	n (%)	p	n (%)	p	n (%)	p
**AHT**						
*Yes*	18 (81.8)	*ns*	15 (68.2)	*ns*	5 (22.7)	*ns*
*No*	148 (79.6)		135 (72.6)		65 (34.9)	
**Diabetes**						
*Yes*	6 (75)	*ns*	5 (62.5)	*ns*	0 (0.0)	*NA*
*No*	160 (80)		145 (72.5)		70 (35)	
**Asthma**						
*Yes*	2 (66.7)	*ns*	2 (66.7)	*ns*	0 (0.0)	*NA*
*No*	164 (80)		148 (72.2)		70 (34.2)	

The proportions of responders against S, RBD, and RBM in COVID-19 samples were determined according to the presence (Yes) or the absence (No) of comorbidities (arterial hypertension (AHT), diabetes, and asthma). Fisher’s exact test was used to compare the proportion of S, RBD, or RBM responders in groups with or without comorbidities.

N, total number of samples; n, number of responders; %, percentage of responders; ns, not significant; NA, not applicable; RBD, receptor-binding domain; RBM, receptor-binding motif.

### Level of Anti-S, Receptor-Binding Domain, and Receptor-Binding Motif Cross-Reacting Antibodies and Active Malaria Infection in the Pre-COVID-19 Malaria Infection Samples

**Figure 7:**
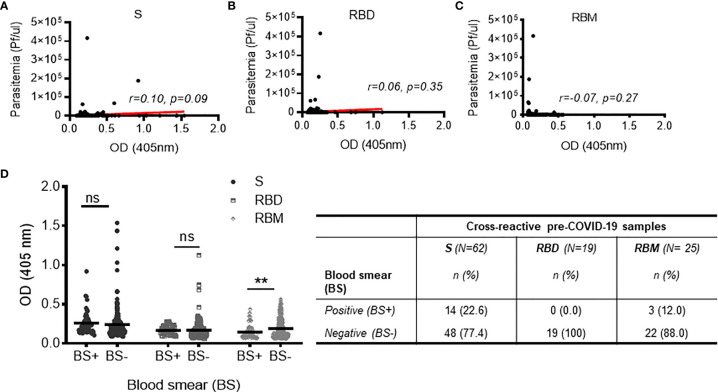
Relationship between cross-reactivity against S, receptor-binding domain (RBD), and receptor-binding motif (RBM) and active malaria infection among pre-COVID-19 donors. **(A–C)** Non-significant correlations of cross-reactive antibodies to S, RBD, or RBM with present malarial infection (i.e., *Plasmodium falciparum* parasitemia in the pre-COVID-19 donor groups). Red lines indicate the best-fit relationship between data points. *p*- and R-values were calculated using the two-tailed Spearman’s rank correlation tests. **(D)** The graph shows no significant variation in cross-reacting antibodies against S and RBD in pre-COVID-19 samples with (blood smear positive (BS+)) or without (blood smear negative (BS−)) present malarial infections; on the other hand, the high level of cross-reactive antibody against RBM was strongly associated (*p* < 0.01) with the absence of malarial infection (BS−). The table shows the proportions of BS+ or BS− cross-reactive samples against S, RBD, and RBM. N, total number of cross-reactive samples; n, number of BS+ or BS− cross-reactive samples. Comparisons of the mean optical density (OD) for BS+ and BS− sample groups were made using the unpaired t-test. **p < 0.01; ns, not significant.

The cross-reactivity of S, RBD, and RBM among the pre-COVID-19 samples from donors living in malaria-endemic areas (Dangassa village) was studied. The antibody OD distribution was similar among the S, RBD, and RBM (*p* > 0.05; **Figure 3**) with respective median antibody ODs (Q1; Q3) of 0.347 (0.269; 0.521), 0.324 (0.308; 0.351), and 0.391 (0.315; 0.467). There was a higher frequency of cross-reactive samples for S (21.9%) than for RBD (6.7%) or RBM (8.8%) (**Figure 3**). In addition, cross-reactive antibodies against all three antigens were present in all age groups; however, they were higher for S and RBM in most age ranges than they were for RBD (**Supplementary Figure 4**). No significant correlation was found between the density of malarial parasitemia and the level of antibodies cross-reacting with S (r = 0.10 *p* = 0.09; **Figure 7A**), RBD (r = 0.06, *p* = 0.35; **Figure 7B**), or RBM (r = −0.07 *p* = 0.27; **Figure 7C**). In contrast, cross-reacting antibodies appeared to be more common in samples without parasitemia (i.e., without active *P. falciparum* infection, or BS− samples), representing 77.4% (42 out of 62), 100% (19 out of 19), and 88% (22 out of 25) of the cross-reactive samples against S, RBD, and RBM, respectively (**Figure 7D**). This correlation is made evident by the fact that BS− samples demonstrated significantly higher mean antibody ODs against RBM than BS+ samples (**Figure 7D**).

## Discussion

Despite the extraordinary breadth of scientific studies on COVID-19, limited data are available from regions where populations are being exposed to additional severe and lethal diseases, such as malaria. This study has demonstrated a high level of seroreactivity for both COVID-19 samples and pre-COVID-19 samples from a malaria-endemic area (Mali) against the SARS-CoV-2 S protein. For the COVID-19 patients (n = 266), most samples reacted with the full-length protein and its internal domain RBD, although responses to the RBM were notably lower. Higher antibody levels at the time of hospital admission were associated with shorter treatment durations for COVID-19. Furthermore, certain comorbidities and the presence of high body weights appeared to be associated with a weaker antibody response to S, RBD, and RBM. The positive response of COVID-19 plasma against different sequence domains (RBD and RBM) of S protein highlights peptide synthesis as an effective vaccine approach, which could ultimately contribute to the mass production of crucial COVID-19 good manufacturing practice (GMP) products (55–57).

Overall, our data demonstrate the importance of RBD—which showed comparable antibody responses (71.9%) to the full-length S protein (80.5%)—as an alternative target for vaccinations and antiviral therapies (58, 59). However it should be noted that we observed a relatively low prevalence of S antibodies (the most prevalent antigen); various other studies observed an antibody response of 95% from their COVID-19 patients (31, 60–64), indicating that our value of 80.5% is lower than expected. This may have been caused by a lack of seroconversion in some patients, as plasma was collected within the first week after hospital admission. According to the literature, at least 11–14 days after the onset of the disease is reported to be necessary to observe an average seroconversion rate of approximately 90%–100% for antibodies (IgM or IgG) against the SARS-CoV-2 S and N proteins (31, 60–69). Future investigations of the antibody dynamics, including in the early (acute) and late (convalescent) phases of COVID-19 infection, may provide more insight into this issue.

Antibody responses to SARS-CoV-2 antigens increased with age but were not associated with gender. Indeed, the S antigen showed a higher antibody level than RBD or RBM across all age groups. The same was observed for RBD as compared to RBM. Some studies have indicated that immunity and COVID-19 infection correlate positively with age (27, 70, 71), while others have suggested that aged patients are more prone to developing an uncontrolled and ineffective immune response, thus increasing disease severity (27, 70, 71). Our data strengthen the argument for inadequate antibody immunity as the cause of higher incidence of hospitalization in elderly patients despite high antibody levels in such groups. Regarding gender, it has been suggested that an immune response to COVID-19 may differ between men and women, thus influencing their ability to recover from a severe infection (72–77). Indeed, in women, higher IgG levels in the early phase and during COVID-19 (72–77) appear to play an essential role in reducing severe disease and mortality (78). However, this study analyzed samples only once, enabling the comparison of antibody levels in mild, severe, and convalescent cases. Still, studies on the dynamics of antibody responses to S, RBD, and RBM—controlling for variables like age and gender—are now necessary. Moreover, it was not possible to determine whether the SARS-CoV-2 antibody levels at hospital admission were correlated with recent exposure to COVID-19, which might explain the benign outcome of the disease in this group of patients.

Concerning treatment duration, patients with stronger responses to S, RBD, or RBM experienced remission in a shorter time period (≤30 days), supporting the idea that S- and RBD-specific antibodies play a crucial role in controlling the severity of SARS-CoV-2 infections. These findings are consistent with other studies that showed that the failure to develop antibodies against SARS-CoV-2 was an essential factor in worsening the disease (79) and was problematic for serodiagnosis tests (30).

This study shows that an accurate assessment of the interactions between preexisting comorbidities and antibody elicitation in the onset of SARS-CoV-2 is essential for existing vaccination strategies and especially to protect those at higher risk from severe forms of COVID-19. Preexisting comorbidities such as diabetes, hypertension, and asthma did not appear to influence antibody response against S and RBD. However, it is interesting that asthma and diabetes seemed to impede the elicitation of antibodies against RBM (the more specific domain for SARS-CoV-2) and that higher body weights appeared to weaken the antibody responses against S in COVID-19 patients. Altogether, these data suggest that preexisting comorbidities—which are associated with disease severity—may be directly impacting the immune responses to SARS-CoV-2 (80–83).

Additionally, our findings imply that even with a lack of specific binding, there is still a high degree of cross-recognition for the SARS-CoV-2 antigens among populations not infected with SARS-CoV-2 living in malaria-endemic areas. Cross-reactive as high as 21.9% against S (highest) is consistent with previous studies, where it reached 17% or even upwards of 20% in malaria-endemic areas (23, 24). This cross-reactivity between malaria and SARS-CoV-2 raises the question of whether other SARS or malaria infections can produce similarly cross-reactive antibodies, playing a role in SARS-CoV-2 infection. In this regard, there is evidence for a cross-neutralization reaction between SARS-CoV and SARS-CoV-2, albeit controversial (25, 84). Malarial infections may also elicit a wide range of immune responses that could also be cross-reactive for COVID-19 antigens (18–22). In addition, antigen cross-reactivity (85, 86) may be due to a non-specific, antigen-independent antibody binding. In pre-COVID-19 volunteers, we observed false positivity against the three antigens in 9.6% to 20.0% of the cross-reactive samples, potentially indicating a non-specific antibody binding. These findings further confirm that anti-SARS-CoV-2 antibody tests may exhibit some false positives, as revealed by ELISA after removing the antigen coating (87, 88). Also, several proteins, present in human plasma at high concentrations—such as albumin (89)—can interfere with the detection of low abundance analytes (90) by increasing background signals and non-specific antibody binding (91).

Moreover, no correlation was found between the cross-recognition of SARS-CoV-2 antigens and current malaria infection. In contrast, the most cross-reactive antibodies were mainly associated with the absence of acute malarial infections, indirectly indicating a protective antibody response to malaria that cross-reacts with SARS-CoV-2. The cross-reactivity is more than likely to occur, since non-specific or poly-specific activation of B cells may occur during or before the process of induction of etiologic antibodies (92–95). Therefore, the coinfection of malaria and COVID-19, their impact on each other (in terms of clinical issues), and the cross-reactivity of COVID-19 antigens with malaria-endemic samples may help to explain the paradox in the incidence of COVID-19 in malaria-endemic areas (20–22, 96–98). Further study is necessary to assess how the coinfection of malaria and SARS-CoV-2 can impact the clinical outcomes of each disease.

In conclusion, the characterization of the individual antibody target domains/epitopes (like RBD and RBM) present in the SARS-CoV-2 S—in both naturally COVID-19 exposed patients and malaria exposed donors without COVID-19 infection—not only would contribute to our understanding of the fine specificity of SARS-CoV-2 antigens and their cross-reactivity observed in these populations but also may offer strategies for designing a second-generation of vaccines. The cross-reactivity of the SARS-CoV-2 antigens was evident in pre-COVID-19 infected samples, as was the impact of protective malarial infection on said cross-reactivity. It can be noted that the early development of high antibody levels against RBD was essential in shortening treatment durations for SARS-CoV-2 infections. Furthermore, factors such as asthma, diabetes, and weight may adversely affect antibody responses to SARS-CoV-2.

## Data Availability Statement

The datasets presented in this study can be found in online repositories. The names of the repository/repositories and accession number(s) can be found in the article/**supplementary material**.

## Ethics Statement

The studies involving human participants were reviewed and approved by the Ethics Committee, EC of the Faculties of Medicine and Odontostomatology, and the Pharmacy of Bamako at the University of Science Technical and Technologies of Bamako, Mali. Written informed consent to participate in this study was provided by the participant’s legal guardian/next of kin.

## Author Contributions

GC and SB designed the experiment. AT, MG, DK, BT, SD, SK, AD, AC, and SB performed most experiments, tests, and analyses. AK, MH, SH, GC, and SB wrote the manuscript. NI, FP, PM, AP, LP, PR, and FE contributed to antigen processing and manuscript revisions. MK, YC, OF, and MD contributed to sample processing and manuscript revisions. All authors read and approved the submitted version.

## Funding

Funding support was received from the University of Sciences, Techniques and Technologies of Bamako (USTTB), Mali.

## Conflict of Interest

The authors declare that the research was conducted in the absence of any commercial or financial relationships that could be construed as a potential conflict of interest.

## Publisher’s Note

All claims expressed in this article are solely those of the authors and do not necessarily represent those of their affiliated organizations, or those of the publisher, the editors and the reviewers. Any product that may be evaluated in this article, or claim that may be made by its manufacturer, is not guaranteed or endorsed by the publisher.

## Footnotes

1 World Health Organization: October 22, 2020|COVID-19: Surveillance, case investigation and epidemiological protocols. https://www.who.int/publications/i/item/considerations-in-the-investigation-of-cases-and-clusters-of-covid-19: [Accessed on October 12, 2021].

2 World Health Organization: March 19, 2020| COVID-19: Infection prevention and control / WASH. https://www.who.int/publications/i/item/10665-331495. [Accessed on October 12, 2021]

3 World Health Organization: Weekly epidemiological update—February 9, 2021. https://www.who.int/publications/m/item/weekly-epidemiological-update---9-february-2021: [Accessed on December 12, 2021].

4 Institut National de Prévoyance Sociale: Rapport de situation COVID-19 au Mali, 10 Janvier 2021 / 04 au 10 Janvier 2021 / N°136. https://covid19-ml.org/: [Accessed January 11, 2021].

5 World Health Organization: COVID-19 vaccines. https://www.who.int/emergencies/diseases/novel-coronavirus-2019/covid-19-vaccines: [Accessed on September 11, 2021].

6 Population-based age-stratified seroepidemiological investigation protocol for coronavirus 2019 (COVID-19) infection, May 26, 2020, version 2.0. https://apps.who.int/iris/handle/10665/332188: [Accessed on September 29, 2021].

7
https://www.ncbi.nlm.nih.gov/sars-cov-2/: [Accessed on September 30, 2021].

8
https://www.epfl.ch/research/facilities/ptpsp/: [Accessed on September 29, 2021].

9
https://www.excellgene.com: [Accessed on September 29, 2021].

## Supplementary Materials

Supplementary Figure 1 Structure and amino acid sequences of the S and RBD proteins, and RBM peptide of SARS-CoV-2. **(A)** Structural features diagram of the SARS-CoV-2 spike (S) protein showing the subunit ectodomains S1 and S2; NTD, the N-terminal domain; RBD, the receptor-binding domain; FP, the fusion peptide; HR1 and HR2, the heptad regions 1 and 2; TM, the transmembrane domain; IC, the intracellular tail, (Yang et al., 2021) [39]. The sequence of ~1250 amino acid (aa) covering the full-length Spike protein is below. The sequence of residues in RBD is shown in green. The full-length sequence of the Spike (S) protein of SARS-CoV-2 is obtained using the BLASTP search program (50, 51). **(B)** The sequence of the SARS-CoV-2 RBD (aa319-529; ~211aa, in green) and several other RBD sequences from different SARS and viruses are provided in parallel for comparison. The portion in magenta, which is more variable than other parts of the RBD domain, is illustrated. * Indicates identical residues; similar residues are green while different ones are red. **(C)** Shows a synthetic peptide sequence (aa436-507; ~72aa) covering the binding segment (RBM, receptor biding motif) of the SARS-CoV-2 RBD. **(D)** Illustrates the 3D structure of SARS-CoV-2 spike (S) protein trimer with an S monomer outlined by blue color, and RBD and RBM in green and magenta, respectively. The other two monomers of S are in grey.

Supplementary Figure 2 Antibody responses against S, RBD and RBM according to gender in COVID-19 and pre-COVID-19 donors. **(A–C)** Show respectively not significant antibody responses (OD) against S **(A)**, RBD **(B)** and RBM **(C)** between male and frmale in COVID-19 samples. Whereas, in pre-COVID-19 samples, the antibody level (cross-reactive antibody) for RBM was significantly higher in female group (p<0.01). The table shows median OD;s and interquartiles (Q1 and Q3) for antibody responses against S, RBD and RBM in COVID-19 and pre-COVID-19 groups. **p≤ 0.01; ns, not significant.

Supplementary Figure 3 Analysis of antibody responses to S, RBD, and RBM according to the presence of multiple comorbid conditions in COVID-19 patients. **(A–C)** Show not significant variation of antibody responses against S, RBD and RBM according to the presence or absense of various comorbidities in COVID-19 patients, respectively. Unlike S and RBD, no association was found between two comorbidities and response to MBR **(C)**. CMB, comorbidity; ns, not significant.

Supplementary Figure 4 Cross-reactivity of S, RBD and RBM according to age group in pre-COVID-19 samples. Cross-reactive antibody levels (in pre-COVID-19 samples) for spike (S) and MBR were comparable, but significantly higher than for MBR in most of the different age groups. Comparison of antibody levels between different antigens in the same age group was determined in unpaired t-test. *p < 0.05; **p < 0.01; ***p < 0.001; NA, not applicable; n, not significant; Age (year), age ranges.

## References

[1] Fehr AR Perlman S. Coronaviruses: An Overview of Their Replication and Pathogenesis. Methods Mol Biol (2015) 1282:1–23. doi: 10.1007/978-1-4939-2438-7_1 25720466 PMC4369385

[2] Peiris JS Guan Y Yuen KY. Severe Acute Respiratory Syndrome. Nat Med (2004) 10(12 Suppl):S88–97. doi: 10.1038/nm1143 PMC7096017 15577937

[3] Zaki AM van Boheemen S Bestebroer TM Osterhaus AD Fouchier RA. Isolation of a Novel Coronavirus From a Man With Pneumonia in Saudi Arabia. N Engl J Med (2012) 367(19):1814–20. doi: 10.1056/NEJMoa1211721 23075143

[4] Gorbalenya AE Baker SC Baric RS de Groot RJ Drosten C Gulyaeva AA. The Species Severe Acute Respiratory Syndrome-Related Coronavirus: Classifying 2019-Ncov and Naming it SARS-CoV-2. Nat Microbiol (2020) 5(4):536–44. doi: 10.1038/s41564-020-0695-z PMC7095448 32123347

[5] Shereen MA Khan S Kazmi A Bashir N Siddique R. COVID-19 Infection: Origin, Transmission, and Characteristics of Human Coronaviruses. J Adv Res (2020) 24:91–8. doi: 10.1016/j.jare.2020.03.005 PMC7113610 32257431

[6] O’Driscoll M Ribeiro Dos Santos G Wang L Cummings DAT Azman AS Paireau J. Age-Specific Mortality and Immunity Patterns of SARS-CoV-2. Nature (2021) 590(7844):140–5. doi: 10.1038/s41586-020-2918-0 33137809

[7] Walsh EE Shin JH Falsey AR. Clinical Impact of Human Coronaviruses 229E and OC43 Infection in Diverse Adult Populations. J Infect Dis (2013) 208(10):1634–42. doi: 10.1093/infdis/jit393 PMC3805243 23922367

[8] Williamson EJ Walker AJ Bhaskaran K Bacon S Bates C Morton CE. Factors Associated With COVID-19-Related Death Using OpenSAFELY. Nature (2020) 584(7821):430–6. doi: 10.1038/s41586-020-2521-4 PMC7611074 32640463

[9] Wu JT Leung K Bushman M Kishore N Niehus R de Salazar PM. Estimating Clinical Severity of COVID-19 From the Transmission Dynamics in Wuhan, China. Nat Med (2020) 26(4):506–10. doi: 10.1038/s41591-020-0822-7 PMC7094929 32284616

[10] Zhu N Zhang D Wang D Li X Yang B Song J. A Novel Coronavirus From Patients With Pneumonia in China. N Engl J Med (2019) 2020(382):727–33. doi: 10.1056/NEJMoa2001017 PMC7092803 31978945

[11] Doumbia S Sow Y Diakite M Lau CY. Coordinating the Research Response to COVID-19: Mali's Approach. Health Res Policy Syst (2020) 18(1):105. doi: 10.1186/s12961-020-00623-8 32943078 PMC7495403

[12] Kouriba B Dürr A Rehn A Sangaré AK Traoré BY Bestehorn-Willmann MS. First Phylogenetic Analysis of Malian SARS-CoV-2 Sequences Provides Molecular Insights Into the Genomic Diversity of the Sahel Region. Viruses (2020) 12(11):1251. doi: 10.3390/v12111251 PMC7692263 33147840

[13] Sagaon-Teyssier L Yattassaye A Bourrelly M Dembélé Keïta B Spire B. The COVID-19 Response Must Integrate People Living With HIV Needs in Sub-Saharan Africa: The Case of Mali. Trop Med Health (2020) 48:41. doi: 10.1186/s41182-020-00228-5 32514230 PMC7268587

[14] Alcázar-Arroyo R Portolés J López-Sánchez P Zalamea F Furaz K Méndez Á.. Rapid Decline of Anti-SARS-CoV-2 Antibodies in Patients on Haemodialysis: The COVID-FRIAT Study. Clin Kidney J (2021) 14(7):1835–44. doi: 10.1093/ckj/sfab048 PMC7989535 34211708

[15] Chi X Yan R Zhang J Zhang G Zhang Y Hao M. A Neutralizing Human Antibody Binds to the N-Terminal Domain of the Spike Protein of SARS-CoV-2. Science (2020) 369(6504):650–5. doi: 10.1126/science.abc6952 PMC7319273 32571838

[16] Nguyen-Contant P Embong AK Kanagaiah P Chaves FA Yang H Branche AR. S Protein-Reactive IgG and Memory B Cell Production After Human SARS-CoV-2 Infection Includes Broad Reactivity to the S2 Subunit. mBio (2020) 11(5):e01991–20. doi: 10.1128/mBio.01991-20 PMC7520599 32978311

[17] Sokal A Chappert P Barba-Spaeth G Roeser A Fourati S Azzaoui I. Maturation and Persistence of the Anti-SARS-CoV-2 Memory B Cell Response. Cell (2021) 184(5):1201–13.e14. doi: 10.1016/j.cell.2021.01.050 33571429 PMC7994111

[18] Chiodini J. COVID-19 and the Impact on Malaria. Travel Med Infect Dis (2020) 35:101758. doi: 10.1016/j.tmaid.2020.101758 32479815 PMC7258844

[19] Hussein MIH Albashir AAD Elawad O Homeida A. Malaria and COVID-19: Unmasking Their Ties. Malar J (2020) 19(1):457. doi: 10.1186/s12936-020-03541-w 33357220 PMC7755982

[20] Iesa MAM Osman MEM Hassan MA Dirar AIA Abuzeid N Mancuso JJ. SARS-CoV-2 and Plasmodium Falciparum Common Immunodominant Regions may Explain Low COVID-19 Incidence in the Malaria-Endemic Belt. New Microbes New Infect (2020) 38:100817. doi: 10.1016/j.nmni.2020.100817 33230417 PMC7674012

[21] Lapidus S Liu F Casanovas-Massana A Dai Y Huck JD Lucas C. Plasmodium Infection Induces Cross-Reactive Antibodies to Carbohydrate Epitopes on the SARS-CoV-2 Spike Protein. medRxiv (2021) 2021.05.10.21256855. doi: 10.1101/2021.05.10.21256855 PMC9778468 36550362

[22] Napoli PE Nioi M. Global Spread of Coronavirus Disease 2019 and Malaria: An Epidemiological Paradox in the Early Stage of A Pandemic. J Clin Med (2020) 9(4):1138. doi: 10.3390/jcm9041138 PMC7230338 32316118

[23] Steinhardt LC Ige F Iriemenam NC Greby SM Hamada Y Uwandu M. Cross-Reactivity of Two SARS-CoV-2 Serological Assays in a Setting Where Malaria Is Endemic. J Clin Microbiol (2021) 59(7):e0051421. doi: 10.1128/jcm.00514-21 33853839 PMC8218747

[24] Yadouleton A Sander AL Moreira-Soto A Tchibozo C Hounkanrin G Badou Y. Limited Specificity of Serologic Tests for SARS-CoV-2 Antibody Detection, Benin. Emerg Infect Dis (2021) 27(1):233–7. doi: 10.3201/eid2701.203281 PMC7774555 33261717

[25] Lv H Wu NC Tsang OT-Y Yuan M Perera RAPM Leung WS. Cross-Reactive Antibody Response Between SARS-CoV-2 and SARS-CoV Infections. Cell Rep (2020) 31(9):107725. doi: 10.1101/2020.03.15.993097 PMC7231734 32426212

[26] Algaissi A Alfaleh MA Hala S Abujamel TS Alamri SS Almahboub SA. SARS-CoV-2 S1 and N-Based Serological Assays Reveal Rapid Seroconversion and Induction of Specific Antibody Response in COVID-19 Patients. Sci Rep (2020) 10(1):16561. doi: 10.1038/s41598-020-73491-5 33024213 PMC7538990

[27] Costagliola G Spada E Consolini R. Age-Related Differences in the Immune Response Could Contribute to Determine the Spectrum of Severity of COVID-19. Immun Inflamm Dis (2021) 9(2):331–9. doi: 10.1002/iid3.404 PMC8014746 33566457

[28] Grzelak L Temmam S Planchais C Demeret C Tondeur L Huon C. A Comparison of Four Serological Assays for Detecting Anti-SARS-CoV-2 Antibodies in Human Serum Samples From Different Populations. Sci Transl Med (2020) 12(559):eabc3103. doi: 10.1126/scitranslmed.abc3103 32817357 PMC7665313

[29] Huang AT Garcia-Carreras B Hitchings MDT Yang B Katzelnick LC Rattigan SM. A Systematic Review of Antibody Mediated Immunity to Coronaviruses: Kinetics, Correlates of Protection, and Association With Severity. Nat Commun (2020) 11(1):4704. doi: 10.1038/s41467-020-18450-4 32943637 PMC7499300

[30] Indenbaum V Koren R Katz-Likvornik S Yitzchaki M Halpern O Regev-Yochay G. Testing IgG Antibodies Against the RBD of SARS-CoV-2 is Sufficient and Necessary for COVID-19 Diagnosis. PloS One (2020) 15(11):e0241164. doi: 10.1371/journal.pone.0241164 33227020 PMC7682882

[31] Long QX Liu BZ Deng HJ Wu GC Deng K Chen YK. Antibody Responses to SARS-CoV-2 in Patients With COVID-19. Nat Med (2020) 26(6):845–8. doi: 10.1038/s41591-020-0897-1 32350462

[32] Perera RA Mok CK Tsang OT Lv H Ko RL Wu NC. Serological Assays for Severe Acute Respiratory Syndrome Coronavirus 2 (SARS-CoV-2), March 2020. Euro Surveill (2020) 25(16):2000421. doi: 10.2807/1560-7917.Es.2020.25.16.2000421 PMC7189648 32347204

[33] Stadlbauer D Tan J Jiang K Hernandez MM Fabre S Amanat F. Repeated Cross-Sectional Sero-Monitoring of SARS-CoV-2 in New York City. Nature (2021) 590(7844):146–50. doi: 10.1038/s41586-020-2912-6 33142304

[34] Arshad AR Bashir I Ijaz F Loh N Shukla S Rehman UU. Is COVID-19 Fatality Rate Associated With Malaria Endemicity? Discoveries (Craiova Romania) (2020) 8(4):e120–0. doi: 10.15190/d.2020.17 PMC7749783 33365386

[35] Rice GI Thomas DA Grant PJ Turner AJ Hooper NM. Evaluation of Angiotensin-Converting Enzyme (ACE), its Homologue ACE2 and Neprilysin in Angiotensin Peptide Metabolism. Biochem J (2004) 383(Pt 1):45–51. doi: 10.1042/bj20040634 15283675 PMC1134042

[36] Zhang H Baker A. Recombinant Human ACE2: Acing Out Angiotensin II in ARDS Therapy. Crit Care (2017) 21(1):305. doi: 10.1186/s13054-017-1882-z 29237475 PMC5729230

[37] Coulibaly D Guindo B Niangaly A Maiga F Konate S Kodio A. A Decline and Age Shift in Malaria Incidence in Rural Mali Following Implementation of Seasonal Malaria Chemoprevention and Indoor Residual Spraying. Am J Trop Med Hyg (2021) 104(4):1342–7. doi: 10.4269/ajtmh.20-0622 PMC8045648 33646974

[38] Wajnberg A Amanat F Firpo A Altman DR Bailey MJ Mansour M. Robust Neutralizing Antibodies to SARS-CoV-2 Infection Persist for Months. Science (2020) 370(6521):1227–30. doi: 10.1126/science.abd7728 PMC7810037 33115920

[39] Yang Y Du L. SARS-CoV-2 Spike Protein: A Key Target for Eliciting Persistent Neutralizing Antibodies. Signal Transduct Target Ther (2021) 6(1):95. doi: 10.1038/s41392-021-00523-5 33637679 PMC7908000

[40] Byrnes JR Zhou XX Lui I Elledge SK Glasgow JE Lim SA. Competitive SARS-CoV-2 Serology Reveals Most Antibodies Targeting the Spike Receptor-Binding Domain Compete for ACE2 Binding. mSphere (2020) 5(5):e00802-20. doi: 10.1128/mSphere.00802-20 PMC7494835 32938700

[41] Khatri I Staal FJT van Dongen JJM. Blocking of the High-Affinity Interaction-Synapse Between SARS-CoV-2 Spike and Human ACE2 Proteins Likely Requires Multiple High-Affinity Antibodies: An Immune Perspective. Front Immunol (2020) 11:570018. doi: 10.3389/fimmu.2020.570018 33042151 PMC7527437

[42] Salvatori G Luberto L Maffei M Aurisicchio L Roscilli G Palombo F. SARS-CoV-2 SPIKE PROTEIN: An Optimal Immunological Target for Vaccines. J Trans Med (2020) 18(1):222. doi: 10.1186/s12967-020-02392-y PMC7268185 32493510

[43] Tai W He L Zhang X Pu J Voronin D Jiang S. Characterization of the Receptor-Binding Domain (RBD) of 2019 Novel Coronavirus: Implication for Development of RBD Protein as a Viral Attachment Inhibitor and Vaccine. Cell Mol Immunol (2020) 17(6):613–20. doi: 10.1038/s41423-020-0400-4 PMC7091888 32203189

[44] Tai W Zhang X He Y Jiang S Du L. Identification of SARS-CoV RBD-Targeting Monoclonal Antibodies With Cross-Reactive or Neutralizing Activity Against SARS-CoV-2. Antiviral Res (2020) 179:104820. doi: 10.1016/j.antiviral.2020.104820 32405117 PMC7219369

[45] Tan TK Rijal P Rahikainen R Keeble AH Schimanski L Hussain S. A COVID-19 Vaccine Candidate Using SpyCatcher Multimerization of the SARS-CoV-2 Spike Protein Receptor-Binding Domain Induces Potent Neutralising Antibody Responses. Nat Commun (2021) 12(1):542. doi: 10.1038/s41467-020-20654-7 33483491 PMC7822889

[46] (WHO) O.m.d.l.S. Guide Pratique Sur L’application Du Règlement Relatif Au Transport Des Matières Infectieuses 2019–2020. Genève: En Vigueur Le 1er Janvier 2019 (2019).

[47] Sun Z Ren K Zhang X Chen J Jiang Z Jiang J. Mass Spectrometry Analysis of Newly Emerging Coronavirus HCoV-19 Spike Protein and Human ACE2 Reveals Camouflaging Glycans and Unique Post-Translational Modifications. Eng (Beijing) (2021) 7(10):1441–51. doi: 10.1016/j.eng.2020.07.014 PMC7456593 32904601

[48] DeLano WLD Delano WL Delano WL Delano WL DeLano WL DeLano W. Pymol: An Open-Source Molecular Graphics Tool. CCP4 Newslett Pro Crystallogr (2002)https://www.scienceopen.com/document?vid=4362f9a2-0b29-433f-aa65-51db01f4962f

[49] Xiong X Qu K Ciazynska KA Hosmillo M Carter AP Ebrahimi S. A Thermostable, Closed SARS-CoV-2 Spike Protein Trimer. Nat Struct Mol Biol (2020) 27(10):934–41. doi: 10.1038/s41594-020-0478-5 PMC7116388 32737467

[50] Altschul SF Madden TL Schäffer AA Zhang J Zhang Z Miller W. Gapped BLAST and PSI-BLAST: A New Generation of Protein Database Search Programs. Nucleic Acids Res (1997) 25(17):3389–402. doi: 10.1093/nar/25.17.3389 PMC146917 9254694

[51] Altschul SF Wootton JC Gertz EM Agarwala R Morgulis A Schäffer AA. Protein Database Searches Using Compositionally Adjusted Substitution Matrices. FEBS J (2005) 272(20):5101–9. doi: 10.1111/j.1742-4658.2005.04945.x PMC1343503 16218944

[52] Ma Z Li P Ji Y Ikram A Pan Q. Cross-Reactivity Towards SARS-CoV-2: The Potential Role of Low-Pathogenic Human Coronaviruses. Lancet Microbe (2020) 1(4):e151. doi: 10.1016/S2666-5247(20)30098-7 33521716 PMC7836609

[53] Ng KW Faulkner N Cornish GH Rosa A Harvey R Hussain S. Preexisting and De Novo Humoral Immunity to SARS-CoV-2 in Humans. Science (2020) 370(6522):1339–43. doi: 10.1126/science.abe1107 PMC7857411 33159009

[54] Lan J Ge J Yu J Shan S Zhou H Fan S. Structure of the SARS-CoV-2 Spike Receptor-Binding Domain Bound to the ACE2 Receptor. Nature (2020) 581(7807):215–20. doi: 10.1038/s41586-020-2180-5 32225176

[55] Corradin G Villard V Kajava AV. Protein Structure Based Strategies for Antigen Discovery and Vaccine Development Against Malaria and Other Pathogens. Endocr Metab Immune Disord Drug Targets (2007) 7(4):259–65. doi: 10.2174/187153007782794371 18220946

[56] Olugbile S Villard V Bertholet S Jafarshad A Kulangara C Roussilhon C. Malaria Vaccine Candidate: Design of a Multivalent Subunit α-Helical Coiled Coil Poly-Epitope. Vaccine (2011) 29(40):7090–9. doi: 10.1016/j.vaccine.2011.06.122 PMC4165486 21803099

[57] Steiner-Monard V Kamaka K Karoui O Roethlisberger S Audran R Daubenberger C. The Candidate Blood-Stage Malaria Vaccine P27A Induces a Robust Humoral Response in a Fast Track to the Field Phase 1 Trial in Exposed and Nonexposed Volunteers. Clin Infect Dis (2019) 68(3):466–74. doi: 10.1093/cid/ciy514 29945169

[58] Jiang S Hillyer C Du L. Neutralizing Antibodies Against SARS-CoV-2 and Other Human Coronaviruses. Trends Immunol (2020) 41(5):355–9. doi: 10.1016/j.it.2020.03.007 PMC7129017 32249063

[59] Okba NMA Müller MA Li W Wang C GeurtsvanKessel CH Corman VM. Severe Acute Respiratory Syndrome Coronavirus 2-Specific Antibody Responses in Coronavirus Disease Patients. Emerg Infect Dis (2020) 26(7):1478–88. doi: 10.3201/eid2607.200841 PMC7323511 32267220

[60] French MA Moodley Y. The Role of SARS-CoV-2 Antibodies in COVID-19: Healing in Most, Harm at Times. Respirology (2020) 25(7):680–2. doi: 10.1111/resp.13852 PMC7280731 32436320

[61] Liu L Liu W Zheng Y Jiang X Kou G Ding J. A Preliminary Study on Serological Assay for Severe Acute Respiratory Syndrome Coronavirus 2 (SARS-CoV-2) in 238 Admitted Hospital Patients. Microbes Infect (2020) 22(4-5):206–11. doi: 10.1016/j.micinf.2020.05.008 PMC7233230 32425648

[62] Lou B Li TD Zheng SF Su YY Li ZY Liu W. Serology Characteristics of SARS-CoV-2 Infection After Exposure and Post-Symptom Onset. Eur Respir J (2020) 56(2):2000763. doi: 10.1183/13993003.00763-2020 32430429 PMC7401320

[63] Suhandynata RT Hoffman MA Kelner MJ McLawhon RW Reed SL Fitzgerald RL. Longitudinal Monitoring of SARS-CoV-2 IgM and IgG Seropositivity to Detect COVID-19. J Appl Lab Med (2020) 5(5):908–20. doi: 10.1093/jalm/jfaa079 PMC7313967 32428207

[64] Ma H Zeng W He H Zhao D Jiang D Zhou P. Serum IgA, IgM, and IgG Responses in COVID-19. Cell Mol Immunol (2020) 17(7):773–5. doi: 10.1038/s41423-020-0474-z PMC7331804 32467617

[65] Gaddi AV Capello F Aluigi L Antignani PL Callegaro A Casu G. The Strategic Alliance Between Clinical and Molecular Science in the War Against SARS-CoV-2, With the Rapid-Diagnostics Test as an Indispensable Weapon for Front Line Doctors. Int J Mol Sci (2020) 21(12):4446. doi: 10.3390/ijms21124446 PMC7352982 32580529

[66] Guo L Ren L Yang S Xiao M Chang D Yang F. Profiling Early Humoral Response to Diagnose Novel Coronavirus Disease (COVID-19). Clin Infect Dis (2020) 71(15):778–85. doi: 10.1093/cid/ciaa310 PMC7184472 32198501

[67] Sun B Feng Y Mo X Zheng P Wang Q Li P. Kinetics of SARS-CoV-2 Specific IgM and IgG Responses in COVID-19 Patients. Emerging Microbes Infect (2020) 9(1):940–8. doi: 10.1080/22221751.2020.1762515 PMC7273175 32357808

[68] Xiang F Wang X He X Peng Z Yang B Zhang J. Antibody Detection and Dynamic Characteristics in Patients With Coronavirus Disease 2019. Clin Infect Dis (2020) 71(8):1930–4. doi: 10.1093/cid/ciaa461 PMC7188146 32306047

[69] Ye Q Zhang T Dezhao L. Potential False-Positive Reasons for SARS-CoV-2 Antibody Testing and its Solution. J Med Virol (2021) 93(7):4242–6. doi: 10.1002/jmv.26937 PMC8250967 33710634

[70] Bajaj V Gadi N Spihlman AP Wu SC Choi CH Moulton VR. Aging, Immunity, and COVID-19: How Age Influences the Host Immune Response to Coronavirus Infections? Front Physiol (2021) 11:571416. doi: 10.3389/fphys.2020.571416 33510644 PMC7835928

[71] Chen Y Klein SL Garibaldi BT Li H Wu C Osevala NM. Aging in COVID-19: Vulnerability, Immunity and Intervention. Ageing Res Rev (2021) 65:101205. doi: 10.1016/j.arr.2020.101205 33137510 PMC7604159

[72] Gadi N Wu SC Spihlman AP Moulton VR. What’s Sex Got to Do With COVID-19? Gender-Based Differences in the Host Immune Response to Coronaviruses. Front Immunol (2020) 11:2147. doi: 10.3389/fimmu.2020.02147 32983176 PMC7485092

[73] Huang B Cai Y Li N Li K Wang Z Li L. Sex-Based Clinical and Immunological Differences in COVID-19. BMC Infect Dis (2021) 21(1):647. doi: 10.1186/s12879-021-06313-2 34225644 PMC8256650

[74] Klein SL Dhakal S Ursin RL Deshpande S Sandberg K Mauvais-Jarvis F. Biological Sex Impacts COVID-19 Outcomes. PloS Pathog (2020) 16(6):e1008570. doi: 10.1371/journal.ppat.1008570 32569293 PMC7307725

[75] Palaiodimos L Kokkinidis DG Li W Karamanis D Ognibene J Arora S. Severe Obesity, Increasing Age and Male Sex are Independently Associated With Worse in-Hospital Outcomes, and Higher in-Hospital Mortality, in a Cohort of Patients With COVID-19 in the Bronx, New York. Metabolism (2020) 108:154262. doi: 10.1016/j.metabol.2020.154262 32422233 PMC7228874

[76] Tadiri CP Gisinger T Kautzy-Willer A Kublickiene K Herrero MT Raparelli V. The Influence of Sex and Gender Domains on COVID-19 Cases and Mortality. CMAJ Can Med Assoc J = J l'Assoc medicale Can (2020) 192(36):E1041–5. doi: 10.1503/cmaj.200971 PMC7504881 32900766

[77] Takahashi T Ellingson MK Wong P Israelow B Lucas C Klein J. Sex Differences in Immune Responses That Underlie COVID-19 Disease Outcomes. Nature (2020) 588(7837):315–20. doi: 10.1038/s41586-020-2700-3 PMC7725931 32846427

[78] Zeng F Dai C Cai P Wang J Xu L Li J. A Comparison Study of SARS-CoV-2 IgG Antibody Between Male and Female COVID-19 Patients: A Possible Reason Underlying Different Outcome Between Sex. J Med Virol (2020) 92(10):2050–4. doi: 10.1002/jmv.25989 PMC7267228 32383183

[79] Cassaniti I Novazzi F Giardina F Salinaro F Sachs M Perlini S. Performance of VivaDiag COVID-19 IgM/IgG Rapid Test is Inadequate for Diagnosis of COVID-19 in Acute Patients Referring to Emergency Room Department. J Med Virol (2020) 92(10):1724–7. doi: 10.1002/jmv.25800 PMC7228409 32227490

[80] Callender LA Curran M Bates SM Mairesse M Weigandt J Betts CJ. The Impact of Pre-Existing Comorbidities and Therapeutic Interventions on COVID-19. Front Immunol (2020) 11:1991. doi: 10.3389/fimmu.2020.01991 32903476 PMC7437504

[81] Guan WJ Liang WH Zhao Y Liang HR Chen ZS Li YM. Comorbidity and its Impact on 1590 Patients With COVID-19 in China: A Nationwide Analysis. Eur Respir J (2020) 55(5):2000547. doi: 10.1183/13993003.00547-2020 32217650 PMC7098485

[82] Singh AK Gillies CL Singh R Singh A Chudasama Y Coles B. Prevalence of Co-Morbidities and Their Association With Mortality in Patients With COVID-19: A Systematic Review and Meta-Analysis. Diabetes Obes Metab (2020) 22(10):1915–24. doi: 10.1111/dom.14124 PMC7361304 32573903

[83] Zhou Y Chi J Lv W Wang Y. Obesity and Diabetes as High-Risk Factors for Severe Coronavirus Disease 2019 (Covid-19). Diabetes Metab Res Rev (2021) 37(2):e3377. doi: 10.1002/dmrr.3377 32588943 PMC7361201

[84] Hoffmann M Kleine-Weber H Schroeder S Krüger N Herrler T Erichsen S. SARS-CoV-2 Cell Entry Depends on ACE2 and TMPRSS2 and Is Blocked by a Clinically Proven Protease Inhibitor. Cell (2020) 181(2):271–280.e8. doi: 10.1016/j.cell.2020.02.052 32142651 PMC7102627

[85] Kenna JG Major GN Williams RS. Methods for Reducing non-Specific Antibody Binding in Enzyme-Linked Immunosorbent Assays. J Immunol Methods (1985) 85(2):409–19. doi: 10.1016/0022-1759(85)90150-4 4078319

[86] Waterboer T Sehr P Pawlita M. Suppression of non-Specific Binding in Serological Luminex Assays. J Immunol Methods (2006) 309(1-2):200–4. doi: 10.1016/j.jim.2005.11.008 16406059

[87] Latiano A Tavano F Panza A Palmieri O Niro GA Andriulli N. False-Positive Results of SARS-CoV-2 IgM/IgG Antibody Tests in Sera Stored Before the 2020 Pandemic in Italy. Int J Infect Dis (2021) 104:159–63. doi: 10.1016/j.ijid.2020.12.067 PMC7834192 33383223

[88] Mboumba Bouassa RS Péré H Tonen-Wolyec S Longo JD Moussa S Mbopi-Keou FX. Unexpected High Frequency of Unspecific Reactivities by Testing Pre-Epidemic Blood Specimens From Europe and Africa With SARS-CoV-2 IgG-IgM Antibody Rapid Tests Points to IgM as the Achilles Heel. J Med Virol (2021) 93(4):2196–203. doi: 10.1002/jmv.26628 33107601

[89] Anderson NL Anderson NG. The Human Plasma Proteome: History, Character, and Diagnostic Prospects*. Mol Cell Proteomics (2002) 1(11):845–67. doi: 10.1074/mcp.r200007-mcp200 12488461

[90] Fountoulakis M Juranville JF Jiang L Avila D Röder D Jakob P. Depletion of the High-Abundance Plasma Proteins. Amino Acids (2004) 27(3):249–59. doi: 10.1007/s00726-004-0141-1 15592754

[91] Dai J Baker GL Bruening ML. Use of Porous Membranes Modified With Polyelectrolyte Multilayers as Substrates for Protein Arrays With Low Nonspecific Adsorption. Anal Chem (2006) 78(1):135–40. doi: 10.1021/ac0513966 16383320

[92] Arneborn P Biberfeld G Forsgren M von Stedingk LV. Specific and non-Specific B Cell Activation in Measles and Varicella. Clin Exp Immunol (1983) 51(1):165–72. PMC1536744 6299636

[93] Biberfeld G Arneborn P Forsgren M von Stedingk LV Blomqvist S. Non-Specific Polyclonal Antibody Response Induced by Mycoplasma Pneumoniae. Yale J Biol Med (1983) 56(5-6):639–42. PMC2590507 6433575

[94] Ratcliffe MJ Julius MH. H-2-Restricted T-B Cell Interactions Involved in Polyspecific B Cell Responses Mediated by Soluble Antigen. Eur J Immunol (1982) 12(8):634–41. doi: 10.1002/eji.1830120803 6982814

[95] Lanzavecchia A Sallusto F. Toll-Like Receptors and Innate Immunity in B-Cell Activation and Antibody Responses. Curr Opin Immunol (2007) 19(3):268–74. doi: 10.1016/j.coi.2007.04.002 17433875

[96] Indari O Baral B Muduli K Prasad Mohanty A Swain N Kumar Mohakud N. Insights Into Plasmodium and SARS-CoV-2 Co-Infection Driven Neurological Manifestations. Biosaf Health (2021) 3(4):230–4. doi: 10.1016/j.bsheal.2021.04.001 PMC8084910 33969285

[97] Onosakponome EO Wogu MN. The Role of Sex in Malaria-COVID19 Coinfection and Some Associated Factors in Rivers State, Nigeria. J Parasitol Res (2020) 2020:8829848. doi: 10.1155/2020/8829848 33354370 PMC7737434

[98] Sardar S Sharma R Alyamani TYM Aboukamar M. COVID-19 and Plasmodium Vivax Malaria Co-Infection. IDCases (2020) 21:e00879. doi: 10.1016/j.idcr.2020.e00879 32665888 PMC7305490

