# Supplementary figures and images for: Seroreactivity of the Severe Acute Respiratory Syndrome Coronavirus 2 Recombinant S Protein, Receptor-Binding Domain, and Its Receptor-Binding Motif in COVID-19 Patients and Their Cross-Reactivity With Pre-COVID-19 Samples From Malaria-Endemic Areas
Source: Front Immunol. 2022 Apr 27;13:856033. doi: 10.3389/fimmu.2022.856033 (PMC9109707; doi:10.3389/fimmu.2022.856033)

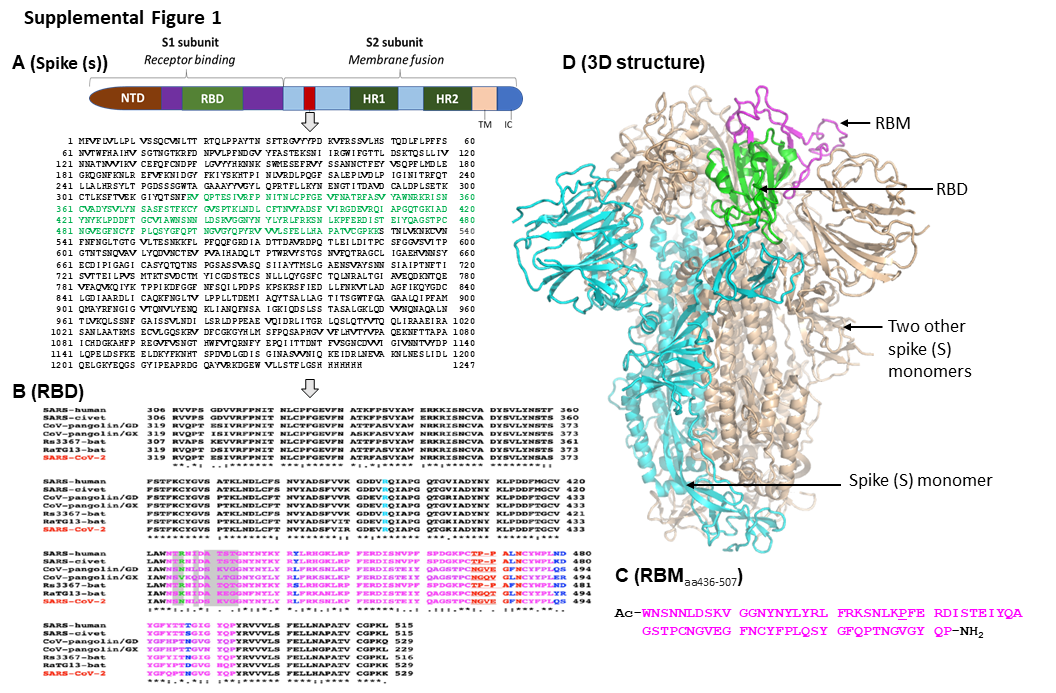

Supplement: Supplementary Figure 1 — Structure and amino acid sequences of the S and RBD proteins, and RBM peptide of SARS-CoV-2. (A) Structural features diagram of the SARS-CoV-2 spike (S) protein showing the subunit ectodomains S1 and S2; NTD, the N-terminal domain; RBD, the receptor-binding domain; FP, the fusion peptide; HR1 and HR2, the heptad regions 1 and 2; TM, the transmembrane domain; IC, the intracellular tail, (Yang et al., 2021) [39]. The sequence of ~1250 amino acid (aa) covering the full-length Spike protein is below. The sequence of residues in RBD is shown in green. The full-length sequence of the Spike (S) protein of SARS-CoV-2 is obtained using the BLASTP search program (50, 51). (B) The sequence of the SARS-CoV-2 RBD (aa319-529; ~211aa, in green) and several other RBD sequences from different SARS and viruses are provided in parallel for comparison. The portion in magenta, which is more variable than other parts of the RBD domain, is illustrated. * Indicates identical residues; similar residues are green while different ones are red. (C) Shows a synthetic peptide sequence (aa436-507; ~72aa) covering the binding segment (RBM, receptor biding motif) of the SARS-CoV-2 RBD. (D) Illustrates the 3D structure of SARS-CoV-2 spike (S) protein trimer with an S monomer outlined by blue color, and RBD and RBM in green and magenta, respectively. The other two monomers of S are in grey. [file Image_1.tiff]

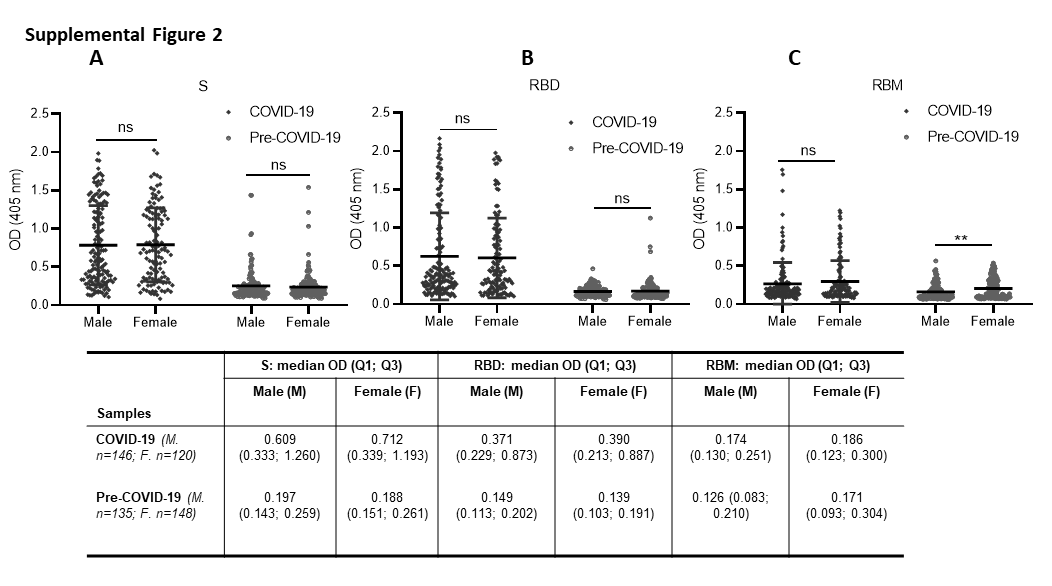

Supplement: Supplementary Figure 2 — Antibody responses against S, RBD and RBM according to gender in COVID-19 and pre-COVID-19 donors. (A–C) Show respectively not significant antibody responses (OD) against S (A), RBD (B) and RBM (C) between male and frmale in COVID-19 samples. Whereas, in pre-COVID-19 samples, the antibody level (cross-reactive antibody) for RBM was significantly higher in female group (p<0.01). The table shows median OD;s and interquartiles (Q1 and Q3) for antibody responses against S, RBD and RBM in COVID-19 and pre-COVID-19 groups. **p≤ 0.01; ns, not significant. [file Image_2.tiff]

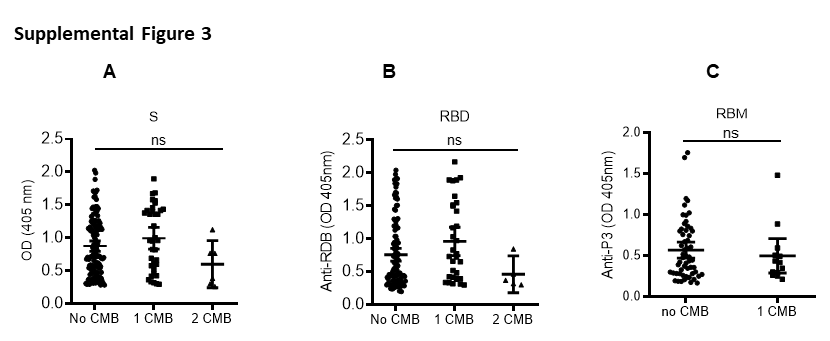

Supplement: Supplementary Figure 3 — Analysis of antibody responses to S, RBD, and RBM according to the presence of multiple comorbid conditions in COVID-19 patients. (A–C) Show not significant variation of antibody responses against S, RBD and RBM according to the presence or absense of various comorbidities in COVID-19 patients, respectively. Unlike S and RBD, no association was found between two comorbidities and response to MBR (C). CMB, comorbidity; ns, not significant. [file Image_3.tiff]

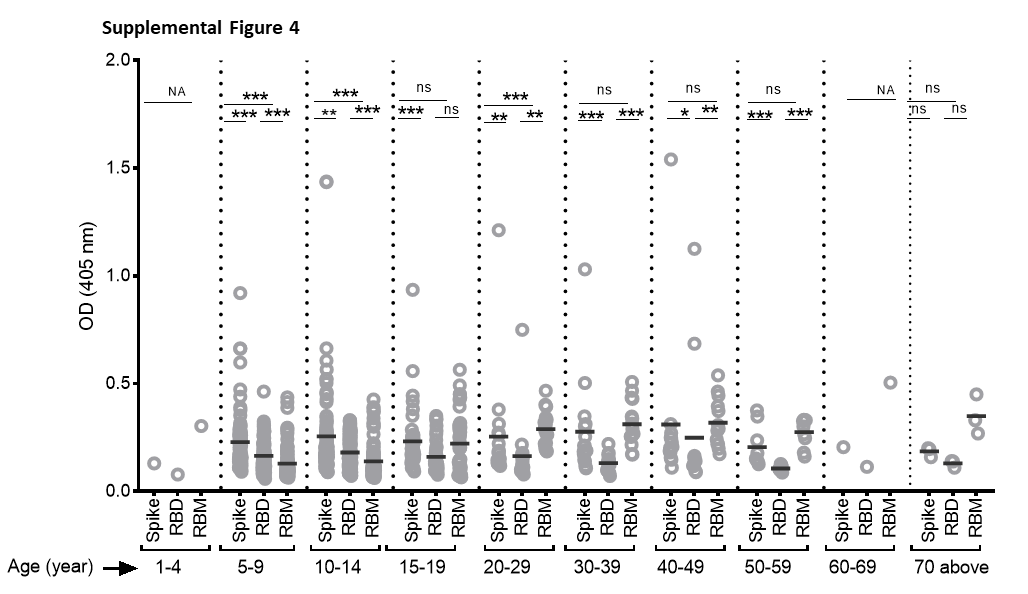

Supplement: Supplementary Figure 4 — Cross-reactivity of S, RBD and RBM according to age group in pre-COVID-19 samples. Cross-reactive antibody levels (in pre-COVID-19 samples) for spike (S) and MBR were comparable, but significantly higher than for MBR in most of the different age groups. Comparison of antibody levels between different antigens in the same age group was determined in unpaired t-test. *p < 0.05; **p < 0.01; ***p < 0.001; NA, not applicable; n, not significant; Age (year), age ranges. [file Image_4.tiff]
